# Beyond the Contract: The Role of Relational and Contractual Governance in Outcome-Based Payment Models

**DOI:** 10.34172/ijhpm.9433

**Published:** 2026-04-06

**Authors:** Maud A. van den Berg, Hilco J. van Elten, Kees T.B. Ahaus

**Affiliations:** ^1^Erasmus School of Health Policy & Management, Erasmus University Rotterdam, Rotterdam, The Netherlands.; ^2^Department of Accounting, Vrije Universiteit Amsterdam, Amsterdam, The Netherlands.

## Introduction

 Governance refers to the mechanisms through which inter-organisational relationships are coordinated and controlled. Organisational research traditionally distinguishes two primary forms: contractual governance, which relies on formal agreements, and relational governance, which emphasises trust and relational norms.^1^ Around 1990, studies often portrayed these governance forms as substitutes, meaning that formal contracts were seen as signals of distrust (limiting the space for relational, trust-based governance), whereas strong relational ties were argued to reduce the need for detailed contractual arrangements.

 In 2002, a new perspective emerged, and scholars began to argue that contractual and relational governance complement rather than replace each other.^2^ On the one hand, well-crafted contracts may foster cooperation, in turn promoting trust and longevity of the relationship. On the other hand, continuity and cooperation can generate contractual refinements, and a certain level of trust may safeguard against unforeseen hazards poorly protected by the contract.^2^ Previous studies have confirmed this relationship and shown a positive link to organisational performance, but longitudinal insights remain limited.^1,3^

 In this viewpoint, we reflect on our experience with the evolution of governance in a healthcare provider-insurer relationship during the design of a new reimbursement model, specifically within the value-based healthcare (VBHC) paradigm. We illustrate how governance mechanisms interact over time in a payment reform trajectory and discuss their potential implications for healthcare system performance.

## Payment Reform and Governance

 VBHC is a strategy aimed at enhancing the quality of care, as measured by outcomes, while also controlling for costs.^4^ In VBHC, reform of reimbursement models is prioritised, as traditional fee-for-service models are primarily based on rewarding volume instead of value.^5^ This is problematic, as healthcare providers experience strong financial disincentives when implementing interventions aimed at enhancing value, such as avoiding high-cost interventions or task shifting from secondary to primary care.^6,7^

 Outcome-based payment models (OBPMs) are suggested as a promising approach. In OBPMs, payment (partly) depends on performance as measured by outcomes of care (the results of and experiences with care processes), aiming to incentivise better outcomes, cost-effective innovation, and reduced overtreatment.^8,9^ However, implementation remains complex and scarce. Beyond technical prerequisites, such as mature information systems and availability of reliable outcome data, OBPMs require changes in how providers and payers govern their relationship.

 Current provider-insurer relationships lean heavily towards contractual governance, and are characterised by a zero-sum orientation, wherein gains for one party are perceived as losses for the other. These dynamics are shaped by competing goals, information asymmetry and limited trust among parties.^10,11^ These practices are reflected in short-term agreements that emphasise cost containment and volume management rather than long-term collaboration or value creation.^12,13^

 In theory, OBPMs aim to create a win–win logic by linking the payment (partly) to outcomes, which benefits both parties: insurers gain from better outcomes at controlled costs, while providers benefit from financial rewards and room for innovation.^8,9^ Achieving this requires providers and insurers to jointly define outcome targets, share data and align around a long-term vision for value creation.^14^ Relational governance mechanisms, such as trust, transparency, flexibility and collaboration, are therefore needed to support the uptake of OBPMs, meaning that the provider-insurer relationship must evolve.^15,16^ However, what such an evolution entails in practice, and how relational and contractual governance interact during this process, is currently unclear.^1-3^

 This viewpoint offers practice-informed reflections on how relational and contractual governance mechanisms can interact over time during the development and implementation of OBPMs. Drawing on experience from practice, we argue that OBPMs can only be developed and implemented through a dynamic, complementary combination of relational and contractual governance, in which each reinforces the other’s strengths and mitigates its limitations. In our view, relational mechanisms enable the required collaboration, flexibility, trust and transparency, but contractual mechanisms remain necessary in providing formal support and safeguarding. Rather than following a fixed or linear sequence, the use of relational and contractual governance mechanisms can be understood as a situational and iterative combination of different mechanisms. By reflecting on this temporal interaction, the viewpoint adds nuance to existing governance concepts and invites further reflection on their application to OBPMs in healthcare.

 The reflections presented here are based on the authors’ direct involvement as academic experts in the development of an OBPM in integrated maternity care in the Netherlands. They include excerpts from meetings and project materials, used illustratively to highlight governance dynamics encountered during the collaboration, and are not presented as empirically analysed qualitative data. Accordingly, the phases described below are illustrative. The project concerned Integrated Maternity Care Organisation Annature Geboortezorg in Breda, the Netherlands. Annature is a network organisation comprising 13 primary-care midwifery practices, six maternity nursing organisations, an Obstetrics & Gynaecology enterprise, and the obstetric department of Amphia Hospital. In this project, Annature collaborated with healthcare insurer CZ, one of the largest insurers in the Breda region. Building on an established trust-based relationship developed through earlier bundled payment reform, both parties jointly explored opportunities to integrate outcomes into the payment model and committed time, resources, and attention to this process. The three illustrative phases presented below show how relational and contractual governance mechanisms interacted and complemented each other throughout the development of the OBPM.

###  Phase 1. Formal Agreements as a Safety Net: Formal Agreements Created Room for Collaboration

 It was the aim of the project to design an OBPM for two redesigned care pathways (ie, the care activities for specific patient populations). In the Netherlands, healthcare providers are contracted by multiple health insurers. Although only one insurer was directly involved in the OBPM design, the support of the other insurers was necessary to ensure accessibility to the new services for all clients in the region. This required collective approval for a temporary payment arrangement to cover the OBPM design period. The healthcare provider drafted a letter-of-intent proposing the temporary payment agreement and details about the OBPM design. In their communication, the provider explicitly emphasised the common goal of the project: *“Measuring outcomes, let alone using outcomes for reimbursement, is largely absent in maternity care. Outcome-based maternity care offers many opportunities to deliver the best possible care to pregnant women and achieve further quality improvements.”*

 The insurers agreed to the temporary payment arrangement on the condition that, in the event of increased costs, the provider’s maximum budget would not be increased. Therefore, the risk of overspending was borne by the provider. The provider accepted this risk due to their belief in the potential impact of the redesign interventions and explicitly stated in the formal agreement that they would bear the risk of overspend. Moreover, the provider promised to be fully transparent about outcomes and costs. Once the letter-of-intent was collectively signed, the project officially commenced.

 This phase exemplifies the interaction between contractual and relational governance. On the one hand, contractual governance facilitated relational governance, as the formal agreement (letter-of-intent) provided a foundation for continuity and cooperation. Drafting and finalising the agreement required interaction between all parties, ensuring a shared understanding of the project’s objectives. On the other hand, relational governance promoted contractual governance. By presenting the initiative as a unifying objective and an opportunity for innovation, the provider secured commitment to collaborate on developing the OBPM.

###  Phase 2. Collective Development of the Contract: Transparency and Co-Creation for Contractual Innovation

 To develop the OBPM, the parties established a taskforce responsible for exploring design opportunities. This group comprised of representatives of both parties and experts in the field concerning VBHC and payment reform. Initially, the taskforce aimed to build a shared understanding of OBPM’s development, as varying interpretations of the model’s purpose and use surfaced. Next, policy frames and legal restrictions were examined. The group then explored different OBPM methods, such as pay-for-performance and shared savings constructions. Finally, cost and outcome data were collected and analysed to identify potential savings and select suitable outcome benchmarks. These insights were addressed in dedicated meetings aimed at synthesising and formalising the OBPM. The provider described the approach as: *“I propose a hybrid model. What I think we are doing now is putting the elements from the task force on the table, and you (insurer) and I (provider) are going to figure out how to make it work. We’re doing this with more insight than usual; it’s more transparent.”*

 Although the parties already had a trusting relationship due to earlier implemented payment model innovation (bundled payment), the provider still felt hesitant to share cost data during the start, fearing negotiation disadvantages by disclosing private information. Nevertheless, the provider acknowledged that full transparency was indispensable for project success, and mentioned: *“[...] If you have already agreed that you want to contract based on outcomes, then the healthcare provider and insurer must form a partnership in which you are transparent and have a good understanding of each other’s interests and the necessary conditions. Yes, my conclusion was that you need [...] to put the cost data on the table.” *Therefore, the provider actively involved the insurer in the cost calculation and illustrated complexities and limitations encountered, which was appreciated by the insurer. To prevent misinterpretations, the provider drafted and shared a disclaimer detailing the cost calculation’s limitations.

 Both governance forms interacted in this phase. The existing trust enabled transparency in cost and outcome data, which was essential for co-creating the contractual elements. Simultaneously, the process revealed that transparency entails risks as well; it can induce fear or perceived risk and thus demands formal safeguards – such as disclaimers – to protect the parties involved.

###  Phase 3. Bridging Conflicting Interests: Relational Governance as Key to Consensus

 During negotiations, tensions surfaced. One of the care pathways resulted in cost savings, creating an opportunity for a shared savings model. A conflict of interest emerged over how to distribute these savings between the provider and the insurer. The provider argued that shared savings constructions must adequately reward providers for pursuing value-enhancing interventions, proportional to the time and effort invested: *“The distribution of savings must do sufficient justice to the fact that healthcare providers make great efforts to improve care delivery, which is labour-intensive, and the distribution of savings should incentivise healthcare providers to continue investing in more appropriate [value-based] care.”*

 Conversely, the insurer insisted the shared savings model to be temporary and the savings distribution to be balanced, as their primary objective is to control healthcare costs. The insurer mentioned: *“As a healthcare insurer, our responsibility is to control healthcare costs. […]. The savings we achieve flow back to our clients in the form of reduced premiums. After all, care is funded by premiums. In our opinion, the scheme you propose is insufficient to meet our social responsibility.”*

 Through intensive dialogue and integrative negotiation (approaching the disagreement as a win-win) the parties developed an allocation model. The provider receives a larger share of savings upon achieving specified outcomes, which gradually decreases over time, shifting the share toward the insurer. The insurer emphasised that this solution was only feasible given the strong existing relationship. The provider remarked: “*A healthcare provider that initiates healthcare improvements and manages to achieve a saving in the process contributes to the social responsibility of reducing healthcare costs. In our opinion, it is important to provide maximum incentives for healthcare providers to do so*.”

 Again, this phase demonstrates how relational and contractual governance interact. The relationship enabled flexibility and adaptability to reach a contractual consensus. Trust and open communication allowed the parties to resolve tensions and reach a workable contractual model that accommodates both parties’ interests.

## Implications

 We argue that the successful development and implementation of OBPM depends on effectively combining contractual and relational governance mechanisms, which interact and reinforce each other over time. In VBHC, this combination supports the win–win logic of OBPMs, benefiting insurers through better outcomes at controlled costs and providers through financial rewards and room for innovation. Relational mechanisms enable the collaboration, flexibility, trust and transparency, while contractual mechanisms provide the necessary formal support and safeguarding. An exclusively contractual approach would fail to manage the complexity of outcome-based contracting: overreliance on control mechanisms, audits and monitoring limits coordination, innovation and flexibility. Conversely, exclusive reliance on relational governance is vulnerable, as trust can take time to build,^17^ and can easily be disrupted by eg, personnel changes or conflicts.^10,18^

 In contexts where trust is low or underdeveloped, the implementation of OBPMs may be premature. Investing in building relational capital is therefore imperative. Initial steps include formulating shared goals and identifying areas of common ground. From there, parties can experiment with collectively interpreting outcome data. This process can reveal specific outcomes requiring improvement, which offers a concrete and relevant entry point for the introduction of an OBPM.

 Building on these implications, we point to “*formal relational contracts*” as a practical governance instrument to combine relational and contractual mechanisms in OBPMs.^19^ Such contract specify “*mutual goals and establishes governance structures to keep the parties’ expectations and interests aligned over the long term.*”^20^ While these contracts are not new, their application to OBPM development in healthcare has received little attention. Implementing such contracts to support the combination of relational and contractual governance is a key strategy for advancing OBPM implementation and the broader VBHC agenda.

## Acknowledgements

 We want to thank Annature research collaboration for collaborating.

## Disclosure of artificial intelligence (AI) use

 Not applicable.

## Conflicts of interest

 Authors declare that they have no conflicts of interest.
